# Data on strains of fungi cultured from baldcypress leaves and gall tissue

**DOI:** 10.1016/j.dib.2017.08.046

**Published:** 2017-09-05

**Authors:** George Washburn, Sunshine A. Van Bael

**Affiliations:** Tulane University, United States

## Abstract

We show the distribution of fungal operational taxonomic units (OTUs) cultured from leaves and galls of baldcypress (*Taxodium distichum*) trees (Washburn and Van Bael, 2017) [1]. We include putative names when possible, guided by the nearest match in the NCBI databank. This data table shows only one representative of each OTU group and it's nearest match in the NCBI databank, along with information about coverage and percent match of the reads. In total there were 144 fungal cultures sequenced, and all sequences were deposited in the NCBI database under accession numbers KY765150–KY765293 (Washburn and Van Bael, 2017) [1].

**Specifications Table**

**Table:** 

Subject area	Biology
More specific subject area	Fungal ecology
Type of data	Table
How data was acquired	Sanger sequencing of the nuclear ribosomal internal transcribed spacer (nrITS) and partial large subunit using primers s ITS1F (5′-CTTGGTCAT TTAGAGGAAGTAA) or ITS5 (5′-GGAAGTAAAAGTCGTAACAAGG) for the forward reaction and LR3 (5′-GGTCCGTGTTTCAAGAC) or ITS4 (5′-TCCTCCGCTTATT GATATGC) for the reverse reaction.
Data format	analyzed
Experimental factors	Comparison of galls that had an insect emerge to galls without insect emergence. Comparison fungi cultured from galls and leaves.
Experimental features	Galls over-wintered and we collected them to analyze their fungal communities and compare them with fungi in leaves.
Data source location	Southeastern Louisiana
Data accessibility	This article, and also in the NCBI database under accession numbers KY765150–KY765293.

**Value of the data**

• These data are the first observations of fungi living in galls of baldcypress midges.
• The sequences and putative identifications can be used for future comparisons in other regions.
• Some of the fungi may be pathogenic on the midges, which may be pests.

## 1 Data

Table 1 describes the number of sequences from fungi isolated from galls and leaves. We compare the fungi isolated from galls with and without insect emergence, as well as displaying their totals. One representative from our 144 cultures for each OTU group is listed in Table 1. We include the percent coverage and match with the nearest match in the NCBI Genbank, along with our matching accession number.

**Table 1:** Putative taxonomic identifications for fungi isolated from galls and leaves of baldcypress.

			**Number of sequences in OTU group**							**NCBI information from search**			
No. OpTaxU	Class	Putative taxonomic ID	No. insect	Live insect	Gall total	Leaf total	Grand total	Isolate no.	GenBank Accession#	Match Accession no.	% Query coverage	% Pairwise identity	Description
3	Sordariomycetes	Sordariomycetes sp.1	0	5	5	26	31	1537	KY765174	JQ760481	100.00%	100.00%	Sordariomycetes sp. genotype 291 isolate FL0837 internal transcribed spacer 1, partial sequence; 5.8S ribosomal RNA gene and internal transcribed spacer 2, complete sequence; and 28S ribosomal RNA gene, partial sequence >gi|387353378|gb|JQ760516.1| Sordariomycetes sp. genotype 291 isolate FL0880 internal transcribed spacer 1, partial sequence; 5.8S ribosomal RNA gene and internal transcribed spacer 2, complete sequence; and 28S ribosomal RNA gene, partial sequence >gi|387354300|gb|JQ761438.1| Sordariomycetes sp. genotype 291 isolate NC0409 internal transcribed spacer 1, partial sequence; 5.8S ribosomal RNA gene and internal transcribed spacer 2, complete sequence; and 28S ribosomal RNA gene, partial sequence >gi|387354402|gb|JQ761540.1| Sordariomycetes sp. genotype 291 isolate NC0548 internal transcribed spacer 1, partial sequence; 5.8S ribosomal RNA gene and internal transcribed spacer 2, complete sequence; and 28S ribosomal RNA gene, partial sequence >gi|387354413|gb|JQ761551.1| Sordariomycetes sp. genotype 291 isolate NC0560 internal transcribed spacer 1, partial sequence; 5.8S ribosomal RNA gene and internal transcribed spacer 2, complete sequence; and 28S ribosomal RNA gene, partial sequence >gi|387354500|gb|JQ761638.1| Sordariomycetes sp. genotype 291 isolate NC0988 internal transcribed spacer 1, partial sequence; 5.8S ribosomal RNA gene and internal transcribed spacer 2, complete sequence; and 28S ribosomal RNA gene, partial sequence >gi|387354700|gb|JQ761838.1| Sordariomycetes sp. genotype 291 isolate NC1197 internal transcribed spacer 1, partial sequence; 5.8S ribosomal RNA gene and internal transcribed spacer 2, complete sequence; and 28S ribosomal RNA gene, partial sequence
6	Sordariomycetes	Pestalotiopsis sp.	17	7	24	0	24	1798	KY765230	EU605881	99.40%	99.60%	Pestalotiopsis sp. AJH25 18S ribosomal RNA gene, partial sequence; internal transcribed spacer 1, 5.8S ribosomal RNA gene, and internal transcribed spacer 2, complete sequence; and 28S ribosomal RNA gene, partial sequence >gi|186695347|gb|EU605882.1| Pestalotiopsis sp. AJH26 18S ribosomal RNA gene, partial sequence; internal transcribed spacer 1, 5.8S ribosomal RNA gene, and internal transcribed spacer 2, complete sequence; and 28S ribosomal RNA gene, partial sequence
4	Sordariomycetes	Sordariomycetes sp.2	0	13	13	7	20	1501	KY765170	EU605881	99.90%	100.00%	Sordariomycetes sp. genotype 510 isolate NC1234 internal transcribed spacer 1, partial sequence; 5.8S ribosomal RNA gene and internal transcribed spacer 2, complete sequence; and 28S ribosomal RNA gene, partial sequence
5	Sordariomycetes	Pestalotiopsis maculiformans	7	4	11	0	11	1794	KY765226	EU552147	99.80%	99.90%	Pestalotiopsis maculiformans culture-collection CBS:122683 18S ribosomal RNA gene, partial sequence; internal transcribed spacer 1, 5.8S ribosomal RNA gene, and internal transcribed spacer 2, complete sequence; and 28S ribosomal RNA gene, partial sequence
7	Dothideomycetes	Botryosphaeria dothidea	3	4	7	0	7	1778	KY765213	AB454278	99.50%	99.60%	Botryosphaeria dothidea genes for ITS1, 5.8S rRNA, ITS2 and 28S rRNA, partial and complete sequence, isolate: MUCC0039
8	Dothideomycetes	Neofusicoccum parvum	1	4	5	0	5	1813	KY765243	KJ657701	100.00%	100.00%	Neofusicoccum parvum strain ALG6 18S ribosomal RNA gene, partial sequence; internal transcribed spacer 1, 5.8S ribosomal RNA gene, and internal transcribed spacer 2, complete sequence; and 28S ribosomal RNA gene, partial sequence
9	Sordariomycetes	Sordariomycetes sp.3	0	5	5	0	5	1824	KY765248	JQ760580	100.00%	100.00%	Sordariomycetes sp. genotype 410 isolate FL0948 internal transcribed spacer 1, partial sequence; 5.8S ribosomal RNA gene and internal transcribed spacer 2, complete sequence; and 28S ribosomal RNA gene, partial sequence >gi|387354311|gb|JQ761449.1| Sordariomycetes sp. genotype 410 isolate NC0420 internal transcribed spacer 1, partial sequence; 5.8S ribosomal RNA gene and internal transcribed spacer 2, complete sequence; and 28S ribosomal RNA gene, partial sequence >gi|387354421|gb|JQ761559.1| Sordariomycetes sp. genotype 410 isolate NC0570 internal transcribed spacer 1, partial sequence; 5.8S ribosomal RNA gene and internal transcribed spacer 2, complete sequence; and 28S ribosomal RNA gene, partial sequence >gi|387354595|gb|JQ761733.1| Sordariomycetes sp. genotype 410 isolate NC1087 internal transcribed spacer 1, partial sequence; 5.8S ribosomal RNA gene and internal transcribed spacer 2, complete sequence; and 28S ribosomal RNA gene, partial sequence >gi|387354628|gb|JQ761766.1| Sordariomycetes sp. genotype 410 isolate NC1121 internal transcribed spacer 1, partial sequence; 5.8S ribosomal RNA gene and internal transcribed spacer 2, complete sequence; and 28S ribosomal RNA gene, partial sequence >gi|387354793|gb|JQ761931.1| Sordariomycetes sp. genotype 410 isolate NC1523 internal transcribed spacer 1, partial sequence; 5.8S ribosomal RNA gene and internal transcribed spacer 2, complete sequence; and 28S ribosomal RNA gene, partial sequence >gi|387354803|gb|JQ761941.1| Sordariomycetes sp. genotype 410 isolate NC1536 internal transcribed spacer 1, partial sequence; 5.8S ribosomal RNA gene and internal transcribed spacer 2, complete sequence; and 28S ribosomal RNA gene, partial sequence
2	Dothideomycetes	Phyllosticta elongata	0	0	0	3	3	1420	KY765153	EU167584	100.00%	100.00%	Phyllosticta elongata strain CBS 114751 small subunit ribosomal RNA gene, internal transcribed spacer 1, 5.8S ribosomal RNA gene, and internal transcribed spacer 2, complete sequence; and large subunit ribosomal RNA gene, partial sequence
11	Unknown	Uncultured fungus 1	2	1	3	0	3	1799	KY765231	KF617937	97.70%	98.60%	Uncultured fungus clone 3268A19 18S ribosomal RNA gene, partial sequence; internal transcribed spacer 1, 5.8S ribosomal RNA gene, and internal transcribed spacer 2, complete sequence; and 28S ribosomal RNA gene, partial sequence
12	Sordariomycetes	Sordariomycetes sp.4	0	0	0	3	3	1500	KY765169	JQ761008	99.90%	100.00%	Sordariomycetes sp. genotype 334 isolate FL1391 internal transcribed spacer 1, partial sequence; 5.8S ribosomal RNA gene and internal transcribed spacer 2, complete sequence; and 28S ribosomal RNA gene, partial sequence
15	Sordariomycetes	Nigrospora oryzae	1	2	3	0	3	1825	KY765249	GQ221861	99.00%	99.40%	Nigrospora oryzae strain NRRL 47510 internal transcribed spacer 1, partial sequence; 5.8S ribosomal RNA gene and internal transcribed spacer 2, complete sequence; and 28S ribosomal RNA gene, partial sequence
1	Unknown	Unidentified fungus 1	0	0	0	2	2	1409	KY765151	KP757567	100.00%	100.00%	Fungal sp. 47 SAB-2015 strain SV661 internal transcribed spacer 1, partial sequence; 5.8S ribosomal RNA gene and internal transcribed spacer 2, complete sequence; and 28S ribosomal RNA gene, partial sequence
10	Unknown	No match available	0	0	0	2	2	1582	KY765182	EU686855	89.50%	92.50%	Fungal endophyte isolate 1417 18S ribosomal RNA gene, partial sequence; internal transcribed spacer 1, 5.8S ribosomal RNA gene, and internal transcribed spacer 2, complete sequence; and 28S ribosomal RNA gene, partial sequence
13	Sordariomycetes	Sordariomycetes sp.5	0	1	1	1	2	1494	KY765166	JQ761863	99.80%	99.90%	Sordariomycetes sp. genotype 333 isolate NC1225 internal transcribed spacer 1, partial sequence; 5.8S ribosomal RNA gene and internal transcribed spacer 2, complete sequence; and 28S ribosomal RNA gene, partial sequence
14	Sordariomycetes	Sordariomycetes sp.6	0	0	0	2	2	1590	KY765185	JQ760654	99.60%	99.80%	Sordariomycetes sp. genotype 422 isolate FL1030 internal transcribed spacer 1, partial sequence; 5.8S ribosomal RNA gene and internal transcribed spacer 2, complete sequence; and 28S ribosomal RNA gene, partial sequence
16	Sordariomycetes	Xylariaceae sp.1	0	0	0	2	2	1400	KY765150	AB741581	99.40%	99.70%	Xylariaceae sp. 4Y-Aj5-1 genes for 18S rRNA, ITS1, 5.8S rRNA, ITS2, 28S rRNA, partial and complete sequence
17	Unknown	No match available	0	1	1	1	2	1857	KY765270	KM519251	92.90%	95.70%	Sordariomycetes sp. genotype caw091 internal transcribed spacer 1, partial sequence; 5.8S ribosomal RNA gene and internal transcribed spacer 2, complete sequence; and 28S ribosomal RNA gene, partial sequence
18	Unknown	Unidentified fungus 2	0	0	0	1	1	1427	KY765156	EU686908	99.70%	99.90%	Fungal endophyte isolate 1595 18S ribosomal RNA gene, partial sequence; internal transcribed spacer 1, 5.8S ribosomal RNA gene, and internal transcribed spacer 2, complete sequence; and 28S ribosomal RNA gene, partial sequence
19	Sordariomycetes	Trichoderma sp.	0	0	0	1	1	1465	KY765159	KC007207	100.00%	100.00%	Trichoderma sp. BESC869k internal transcribed spacer 1, partial sequence; 5.8S ribosomal RNA gene and internal transcribed spacer 2, complete sequence; and 28S ribosomal RNA gene, partial sequence
20	Unknown	No match available	0	0	0	1	1	1488	KY765163	KP263093	91.60%	94.70%	Hypocreales sp. P30042SNA1CC1087 internal transcribed spacer 1, partial sequence; 5.8S ribosomal RNA gene and internal transcribed spacer 2, complete sequence; and 28S ribosomal RNA gene, partial sequence
21	Sordariomycetes	Sordariomycetes sp.7	0	0	0	1	1	1513	KY765172	JQ761712	99.80%	99.90%	Sordariomycetes sp. genotype 510 isolate NC1066 internal transcribed spacer 1, partial sequence; 5.8S ribosomal RNA gene and internal transcribed spacer 2, complete sequence; and 28S ribosomal RNA gene, partial sequence
22	Unknown	No match available	0	0	0	1	1	1549	KY765176	GU055669	95.40%	97.20%	Uncultured Fusarium clone NG_R_C10 18S ribosomal RNA gene, partial sequence; internal transcribed spacer 1, 5.8S ribosomal RNA gene, and internal transcribed spacer 2, complete sequence; and 28S ribosomal RNA gene, partial sequence
23	Unknown	Unidentified fungal endophyte	0	0	0	1	1	1558	KY765177	KF435424	98.70%	99.30%	Fungal endophyte culture-collection STRI:ICBG-Panama:TK117 18S ribosomal RNA gene, partial sequence; internal transcribed spacer 1, 5.8S ribosomal RNA gene, and internal transcribed spacer 2, complete sequence; and 28S ribosomal RNA gene, partial sequence
24	Sordariomycetes	Diaporthe sp.	0	0	0	1	1	1586	KY765183	KR094451	99.30%	99.60%	Diaporthe sp. G360 18S ribosomal RNA gene, partial sequence; internal transcribed spacer 1, 5.8S ribosomal RNA gene, and internal transcribed spacer 2, complete sequence; and 28S ribosomal RNA gene, partial sequence
25	Sordariomycetes	Xylariaceae sp.2	0	0	0	1	1	1673	KY765197	AB741599	100.00%	100.00%	Xylariaceae sp. 4Y-Dd2-5 genes for 18S rRNA, ITS1, 5.8S rRNA, ITS2, 28S rRNA, partial and complete sequence
26	Dothideomycetes	Phyllosticha sp.1	0	0	0	1	1	1709	KY765202	AB454329	99.40%	99.60%	Phyllosticta sp. MUCC0158 genes for ITS1, 5.8S rRNA, ITS2 and 28S rRNA, partial and complete sequence, isolate: MUCC0158
27	Sordariomycetes	Colletotrichum aeschynomenes	0	0	0	1	1	1734	KY765207	JX131331	100.00%	100.00%	Colletotrichum aeschynomenes isolate Clar-5A 18S ribosomal RNA gene, partial sequence; internal transcribed spacer 1, 5.8S ribosomal RNA gene, and internal transcribed spacer 2, complete sequence; and 28S ribosomal RNA gene, partial sequence
28	Sordariomycetes	Sordariomycetes sp.8	0	1	1	0	1	1784	KY765217	JQ760187	99.70%	99.80%	Sordariomycetes sp. genotype 309 isolate FL0462 internal transcribed spacer 1, partial sequence; 5.8S ribosomal RNA gene and internal transcribed spacer 2, complete sequence; and 28S ribosomal RNA gene, partial sequence
29	Sordariomycetes	Sordariomycetes sp.9	0	1	1	0	1	1789	KY765222	JQ761634	100.00%	100.00%	Sordariomycetes sp. genotype 573 isolate NC0984 internal transcribed spacer 1, partial sequence; 5.8S ribosomal RNA gene and internal transcribed spacer 2, complete sequence; and 28S ribosomal RNA gene, partial sequence >gi|387354549|gb|JQ761687.1| Sordariomycetes sp. genotype 573 isolate NC1040 internal transcribed spacer 1, partial sequence; 5.8S ribosomal RNA gene and internal transcribed spacer 2, complete sequence; and 28S ribosomal RNA gene, partial sequence >gi|387354699|gb|JQ761837.1| Sordariomycetes sp. genotype 573 isolate NC1196 internal transcribed spacer 1, partial sequence; 5.8S ribosomal RNA gene and internal transcribed spacer 2, complete sequence; and 28S ribosomal RNA gene, partial sequence
30	Dothideomycetes	Aureobasidium sp.	1	0	1	0	1	1814	KY765244	FN665417	99.40%	99.60%	Aureobasidium sp. RBF-3B2 18S rRNA gene (partial), ITS1, 5.8S rRNA gene, ITS2 and 26S rRNA gene (partial), isolate RBF-3B2
31	Eurotomycetes	Periconia sp.	0	1	1	0	1	1823	KY765247	HQ130695	99.30%	99.50%	Periconia sp. WF138 18S ribosomal RNA gene, partial sequence; internal transcribed spacer 1, 5.8S ribosomal RNA gene, and internal transcribed spacer 2, complete sequence; and 28S ribosomal RNA gene, partial sequence
32	Eurotomycetes	Penicillium sp	1	0	1	0	1	1833	KY765250	HM469409	99.50%	99.70%	Penicillium sp. 6 JJK-2011 18S ribosomal RNA gene, partial sequence; internal transcribed spacer 1, 5.8S ribosomal RNA gene, and internal transcribed spacer 2, complete sequence; and 28S ribosomal RNA gene, partial sequence
33	Sordariomycetes	Sordariomycetes sp.10	0	1	1	0	1	1864	KY765275	JQ761854	99.90%	100.00%	Sordariomycetes sp. genotype 293 isolate NC1214 internal transcribed spacer 1, partial sequence; 5.8S ribosomal RNA gene and internal transcribed spacer 2, complete sequence; and 28S ribosomal RNA gene, partial sequence

## 2 Experimental design, materials and methods

We amplified the nuclear ribosomal internal transcribed spacer (nrITS) and partial large subunit using primers s ITS1F (5′-CTTGGTCAT TTAGAGGAAGTAA) or ITS5 (5′-GGAAGTAAAAGTCGTAACAAGG) for the forward reaction and LR3 (5′-GGTCCGTGTTTCAAGAC) or ITS4 (5′-TCCTCCGCTTATT GATATGC) for the reverse reaction. Our cycling protocol for amplification followed [2].

After visualization confirmation with SYBR on 1% agarose gels, we sent the PCR products to Beckman Coulter Genomics (Boston, MA) for Sanger sequencing. We sent 164 PCR products from individual cultures of gall or foliar fungi. Due to low quality in the sequences, 20 samples were removed before analyses, leaving 144 sequences. The sequences were assembled and edited in Sequencher v5.0 (Gene Codes Corp., Ann Arbor, MI) with support from Mesquite. Operational taxonomic units (OTUs) were formed from sequences assembled based on 97% similarity (Table 1). These OTUs were compared to the NCBI archives through BLAST searches to assign putative taxonomic identities based on the sequence similarity. For each OTU described in Table 1 of [1], we include the accession and strain number of a representative strain from our collection, the accession number of the nearest match in the NCBI databank, percent identity and query cover information. Voucher cultures were archived in the Van Bael laboratory at Tulane University and are available from the author by request.
