# Supplementary material for: Data on strains of fungi cultured from baldcypress leaves and gall tissue
Source: Data Brief. 2017 Sep 5;14:793–803. doi: 10.1016/j.dib.2017.08.046 (PMC5601304; doi:10.1016/j.dib.2017.08.046)
Supplement: Supplementary file 1 — Supplementary material [file mmc1.docx]

Conflict of interest form could not be located. Please send it to me.
